# A new therapy (MP29-02*) effectively targets the entire seasonal allergic rhinitis symptom complex

**DOI:** 10.1186/2045-7022-3-S2-P45

**Published:** 2013-07-16

**Authors:** David Price, Jean Bousquet, Claus Bachert, Wytske Fokkens, Glenis Scadding, Ullrich Munzel, Peter Hellings

**Affiliations:** 1 University of Aberdeen, Aberdeen, UK; 2 Hopital Arnaud de Villeneuve University Hospital, Montpellier, France; 3 Ghent University Hospital, Dept of Otorhinolaryngology, Ghent, Belgium; 4 Academic Medical Center, Dept of Otorhinolaryngology, Amsterdam, the Netherlands; 5 The Royal National Throat, Nose and Ear Hospital, London, UK; 6 Meda Pharma, Biostatistics & Market Access, Bad Homburg, Germany; 7 University Hospitals Leuven, Dept of Otorhinolaryngology, Head and Neck Surgery, Leuven, Belgium

## Background

Over 90% of allergic rhinitis (AR) patients have ocular symptoms during the pollen season, but these are routinely neglected and under-treated. New treatment options should provide relief not only from all nasal symptoms, but all ocular symptoms too, since patients frequently present with symptoms from both nasal and ocular origin. The reflective total of 7 symptom scores (rT7SS) is an endpoint measuring the entire AR symptom complex. It comprises both the reflective total nasal symptom score (rTNSS) and the reflective total ocular symptom score in one global score (max score=42).

## Objective

To assess the efficacy of MP29-02* (a novel intranasal formulation of azelastine hydrochloride [AZE] and fluticasone propionate [FP]) in providing relief from the entire AR symptom complex compared to commercially available intranasal AZE or FP nasal sprays and placebo, in patients with seasonal AR (SAR).

## Methods

610 patients (>=12 years old) with moderate-to-severe SAR were randomized into this double-blind, placebo-controlled, 14-day, parallel-group trial to MP29-02*, commercially-available AZE or FP nasal sprays, and placebo (all given as 1 spray/nostril bid [total daily doses: AZE = 548 µg; FP=200 µg]. The primary efficacy variable was change from baseline in rTNSS (AM +PM), over 14-days. Change from baseline in rT7SS was assessed post-hoc via an analysis of covariance.

## Results

MP29-02* most effectively treated the entire rhinitis symptom complex, reducing the rT7SS from baseline by -8.74 compared to -6.05 for FP (Diff: -2.69; 95% CI: -4.33, -1.06; p=0.0013), -5.83 for AZE (Diff -2.91; 95% CI: -4.52, -1.31; p=0.0004) and -3.55 for placebo (Diff -5.19; 95% CI: -6.71, -3.68; p<0.0001). The relative difference was 52% to FP and 56% to AZE, making MP29-02* twice as effective as either firstline therapy. This benefit was observed during the first day of treatment and was sustained over the entire course of treatment.

## Conclusion

Compared to currently available first-line therapy for AR, MP29-02* most effectively treats the entire rhinitis symptom complex, comprising the most commonly reported nasal and ocular symptoms. Such a universal treatment option should preclude the need for concomitant eye drops, and may be considered the drug of choice for AR management.

*Dymista

